# Patterns of Suicide and Other Trespassing Fatalities on State-Owned Railways in Greater Stockholm; Implications for Prevention

**DOI:** 10.3390/ijerph9030772

**Published:** 2012-03-05

**Authors:** Helena Rådbo, Ragnar Andersson

**Affiliations:** Department of Health and Environmental Sciences, Karlstad University, Karlstad 65188, Sweden; Email: ragnar.andersson@kau.se

**Keywords:** suicide, railway, railroad, suicide prevention, railway safety

## Abstract

Each year, approximately 80–100 people are killed on state-owned railways due to train-person collisions in Sweden. Underlying causes are suicide and accidents; suicide constituting a vast majority. Earlier Swedish studies at a national level revealed a relation between population density and incident frequency, however, with places of occurrence often located to the outskirts of cities some distance away from station areas where victims can await approaching trains in seclusion. The aim of this study was to investigate whether this national pattern also applies to larger urban areas such as greater Stockholm, and to discuss preventative implications based on these observations. All registered incidents (N = 41) where people were hit or run-over by trains with a fatal outcome over the four-year period 2005–2008 were investigated. Results deviating from the national pattern include that most incidents occur at station areas, and that most victims enter the tracks from platforms. Passing express trains appear to be overrepresented, compared to commuter trains. Due to a low number of cases, our observations must be interpreted with caution. However, they imply that preventative measures in this type of area should focus on platform safety foremost, especially protection against rapid trains passing by station areas.

## 1. Introduction

We have previously investigated train-person collisions on the state-owned Swedish rail network at the national level as a basis for systems-oriented prevention [1,2,3,4]. Systems-oriented measures refer to both technical and environmental changes, as well as efforts targeting operation, planning, and management of the system. We found an annual national toll of about 80–100 people being killed from such incidents, of which about 75% are classified as suicide by the police. We also found that a majority of the suicide victims appeared to have been waiting on or nearby tracks for a period of time before the train arrived, potentially leaving time for early detection and prompt intervention. With regard to place, we found that most collisions occur in densely populated areas, however, often some distance away from stations and platforms at more hidden spots and usually in the vicinity of the victim’s residence. Incidents were concentrated to urban areas, especially the three major cities; Stockholm, Gothenburg and Malmoe. This result is also important from a preventive point of view, since it means that measures can be prioritized to limited geographical areas. When comparing suicidal and accidental cases, we found differences with regard to individual and temporal characteristics, but similarities in injury mechanisms and possible countermeasures. This lead to the conclusion that prevention of railway suicide can benefit from a spectrum of well-known accident preventative strategies and should therefore become an integral part of regular railway safety work. This view was broadly accepted by practitioners as well, when discussing our findings in focus groups.

The international literature in this field is limited. Several studies describe the frequency and characteristics of railway suicide in different countries [5,6,7,8,9,10,11,12,13], but only few of them explore systems-oriented preventative possibilities in more detail [14,15], mostly with reference to underground systems [16,17]. Our findings on victims awaiting trains on site and the nearness between place of occurrence and place of residence are in line with findings from other studies [5]. A special branch of studies refer to “hot spots”, especially their nearness to psychiatric institutions [18,19]. From our national studies we have not been able to identify geographical clustering patterns in that respect, possibly as a result of a broad transition to non-institutional psychiatric care in the Swedish society. Instead we have chosen to look deeper into the urban patterning of train-person collisions against the background of earlier results.

The aim of the current study is to analyze train-person collision fatalities on state-owned rail networks in the Stockholm region with regards to place as well as temporal, demographical, behavioral and technical patterns relevant for safety work. Special attention is paid to patterns deviating from the national picture, implying special conditions and needs in larger urban settings.

## 2. Materials and Methods

This study is a total investigation (census; no sampling) of all train-person collisions on the dominating state-owned rail network in greater Stockholm (defined below) occurring during the years 2005–2008 and registered by the Swedish Transport Administration (STA). STA is the national agency responsible for the state-owned rail network in Sweden, including its infrastructural safety. Public rail transportation to a lesser extent also exists on private and municipal rail systems (underground, tram and a few commuter lines) within the same geographical area. This study was conducted in collaboration with STA with a special focus on networks under their own governance. Incidents on these systems are therefore not included.

### 2.1. Setting

Greater Stockholm rail district is divided into five geographical sections; North (N), South-East (SE), South-West (SW), West (W), and Central (C). The sections differ slightly in terms of number and length of tracks, train categories, and intensity. The north section and parts of the central section are operated by frequent express trains to and from the region’s main international airport, as well as commuter and long-distance trains. 

### 2.2. Data Sources

Data from two sources have been used for this study, both administered by STA:

*Synergi* is an incident reporting system in operation since 2005. Records on train-person collisions include free-text descriptions on the event, information on when and where the collision took place, rail-related companies involved, the consequences, causes and immediate measures taken, as well as a classification of the type of event based on police reports. Data on sex, age and outcome (deceased or injured) are also included. More comprehensive investigation reports compiled by STA investigators with references to police observations on the same incidents are attached in most cases. 

*STRIX* is a mobile track status monitoring system which includes video recording, allowing visual observation of tracks, aerial lines and the nearest surroundings, without physical presence on site.

### 2.3. Data Analysis

Quantitative data were extracted from the sources above by the main author, based on coded and free-text information, according to a variable structure developed in earlier studies [1,2,3]. Variables of interest include background victim data (age, sex), type of train, place characteristics (location, type of place, surroundings, barrier status), and crash details (the victim’s pre-crash behavior, injury mechanism, intent as defined by the police, time of occurrence). Rudimentary descriptive analyses were performed with the SPSS Statistics 19 program package.

## 3. Results

### 3.1. Intent, Age and Sex

A total of 47 cases of train-person collisions were recorded during the period, of which 41 were fatal. These are further presented in this study. The cause of collision among these was in 30 cases classified as suicide by the police, and in the remaining 11 cases as accidents. 

Twenty-nine of the victims were male and 12 female. The age span was between 15 and 91 years, with most victims in the ages 40–59 for both suicide and all cases (Table 1). Mean and median ages for suicide were 40 and 42, respectively, while for accidents the corresponding ages were 37 and 24 years. 

**Table 1:** Fatal train-person collisions by sex, age, and injury intent on railway property in the greater Stockholm area, 2005–2008.

Variables		Type of injury event		
		Suicide	Accident	Total
**Sex and age**	Male	20	9	29
	Female	10	2	12
	Total	30	11	41
	0–19	3	2	5
	20–39	9	5	14
	40–59	14	3	17
	60–79	3	0	3
	80–	0	1	1
	Unknown	1	0	1
	Total	30	11	41

### 3.2. Time of Occurrence

Nine cases occurred in 2005, 11 in 2006, 17 in 2007 and 10 in 2008, resulting in an average of one incident per month in this district including the six non-fatal cases. Table 2 shows the aggregate distribution by month, day of the week, and hour. Suicide cases occurred mostly on weekdays, while accidents were more common at weekends. Similarly, suicides were more common in the daytime/afternoon, while accidents primarily occurred later at night.

**Table 2:** Fatal train-person collisions by time of occurrence (month, day and hour) and type of injury on railway property in the greater Stockholm area 2005–2008.

Variables		Type of injury event		
		Suicide	Accident	Total
**Month**	Jan–March	9	5	14
	April–June	9	1	10
	July–Sept	7	2	9
	Oct–Dec	5	3	8
	Total	30	11	41
**Day of the week**	Monday	2	1	3
	Tuesday	9	0	9
	Wednesday	5	0	5
	Thursday	5	3	8
	Friday	3	1	4
	Saturday	3	4	7
	Sunday	3	2	5
	Total	30	11	41
**Hour**	00–06	2	3	5
	06–12	7	3	10
	12–18	16	1	17
	18–24	5	4	9
	Total	30	11	41

### 3.3. Place Characteristics and Pre-Crash Behavior

Among the victims in this study platforms were commonly used to enter track areas (Table 3). It was not possible to determine the duration of the victims’ presence on the platforms from our sources. In several cases, however, victims were observed in advance, but did not distinguish themselves enough to prompt actions from bystanders.

**Table 3:** Fatal train-person collisions by place, destination and pre-crash behavior on railway property in the greater Stockholm area, 2005–2008.

Variables	Type of injury event		
	Suicide	Accident	Total
**Type of place**			
Platform area	16	8	24
Station area outside platform	9	1	10
Level crossing	1	2	3
Other and unknown	4	0	4
Total	30	11	41
**Track section**			
Central part [C]	5	4	9
North [N]	11	2	13
West [W]	4	3	7
South west [SW]	5	2	7
South east [SE]	5	0	5
Total	30	11	41
**The victim’s pre-crash behavior**			
Standing/walking on tracks	11	2	13
Lying/sitting on tracks	9	0	9
Jumping/running in front of the train	9	0	9
Other	0	2	2
Taking short cuts	0	7	7
Missing	1	0	1
Total	30	11	41

Among the suicides, 16 cases were hit adjacent to platforms, while in nine cases collision occurred at places some distance from the platform, but probably accessed from the platforms as well. One case was hit close to a level crossing and the remaining four cases lack detailed information in this respect.

With regards to the accidents, eight cases occurred adjacent to platforms, but with deviating activity patterns compared to suicides. These included attempting to take a shortcut between platforms and falling from the platform.

### 3.4. Track Sections and Train Categories

Most cases occurred within the northern track section (13 cases), and least in the south-east section (five cases). Train categories involved include local commuter trains (19 cases), long distance fast trains (seven cases) and airport express trains (14 cases). At the north section, airport express trains were involved in 11 out of 13 incidents in total; nine suicides and two accidents. See Table 4.

**Table 4:** Fatal train-person collisions by track sections and train categories on railway property in the greater Stockholm area, 2005–2008.

Variables		Train categories				
		Commuter train	Long distance fast train	Airport express train	Missing	Total
**Track section**	C	5	1	3	0	9
	SW	6	1	0	0	7
	N	0	2	11	0	13
	W	4	3	0	0	7
	SE	4	0	0	1	5
	Total	19	7	14	1	41

## 4. Discussion

This study was a total investigation of all registered train-person collisions with fatal outcome on state-owned rail networks in the greater Stockholm metropolitan area over a four year period 2005–2008. The limited material does not allow detailed statistical analyses and conclusions such as comparisons of risk between track sections, but points nevertheless to important patterns and tendencies relevant for preventative considerations. Moreover, all cases are carefully investigated by STA’s own investigator as well as the main author of this paper which brings the methodological approach of this study close to a qualitative multiple case study [20]. For the sake of comparison with national findings, however, we decided to present our results quantitatively. Due to the limited size of our material, our results should be interpreted with caution. 

The proportion of suicides and accidents in our material are similar to earlier findings at national level with suicide being the overriding problem [1]. Sex and age distributions are also similar to other studies [1,5,9,11,12,13]. Most victims, in both categories, are men and usually middle-aged in suicides while younger in accidents. Also, temporal distributions show similarities with earlier national findings and findings from other countries [1,21,22]. Suicide is typically a weekday and daytime problem while accidents are more common at night and weekends.

Information on the victim’s pre-crash behavior usually originates from train-drivers’ and bystanders’ testimonies. In *suicide*, victims are observed awaiting trains at the platform, standing/walking near the edge, or, less commonly, wandering about at level crossings, however usually not conspicuously enough to prompt action. In one third of the cases the victim jumped in front of the train. In only one case, an SOS alarm was triggered, though not early enough to prevent the incident. At national level, awaiting trains for some time on or near tracks at crossings or in secluded areas is a more common pattern [1,3]. The importance of learning from the victims’ pre-crash behavior is also highlighted by others [23]. Among *accidents*, the corresponding information reveals other scenarios. Victims were observed walking in the wrong direction, or taking shortcuts through broken fences, under closed gates or across tracks between platforms.

The most striking finding, which clearly deviates from the national pattern, is the choice of platforms as entrances to the track area among suicide victims. This could possibly be explained by better fencing and fewer unprotected level crossings compared to the country as a whole, leaving fewer options to access the track area but from platforms. Whether this in turn results in fewer incidents than otherwise expected, or merely a spatial redistribution of cases, is something we cannot comment on from our results. It is claimed by others, however, that impeding accessibility to means of suicide also has an absolute preventative effect [24]. The existence of frequent express trains passing near platforms at high speeds, representing an easily accessible and very potent means of suicide, might also increase the attractiveness of platforms among suicide attempters in this urban area. 

All trains involved were passenger trains, and commuter trains were most affected. However, these trains are also the most common in the area which may explain their larger involvement. Compared to frequency at different track sections, the airport express line appears considerably overrepresented. On the north section, commuter trains are nearly twice as frequent as airport trains but are involved in much fewer incidents. All incidents with airport trains occurred when trains passed station areas without stopping. 

Taking all results into consideration, we believe that preventative efforts should concentrate on separating people from trains in motion, especially those passing by at high speeds, either by physical barriers or by redirecting passing trains to less accessible tracks. Identifying suicide attempters at crowded platforms may be a difficult task, but effective surveillance or sensor systems could clearly contribute to improved safety in cases when persons are attempting to trespass prohibited areas before trains arrive or pass.

## 5. Conclusions

The pattern of train-person collisions in the greater Stockholm metropolitan area differs from the national pattern in one important respect. Most incidents occur in the immediate vicinity of platforms, indicating that more attention should be devoted to platform safety compared to other parts of the country. Express trains, passing by at high speeds, represent a special concern.
